# Changes in Anxiety-Related Behaviors, Voiding Patterns, and Urinary Bladder Contractile Properties in Male Mice Exposed to Water Avoidance Stress for 1 Day and 28 Days

**DOI:** 10.3390/biology13090707

**Published:** 2024-09-09

**Authors:** Sarunnuch Sattayachiti, Panida Chumpong, Seree Niyomdecha, Dania Cheaha, Nipaporn Konthapakdee

**Affiliations:** 1 Division of Health and Applied Sciences, Faculty of Science, Prince of Songkla University, Songkhla 90110, Thailand; 2 Division of Biological Science, Faculty of Science, Prince of Songkla University, Songkhla 90110, Thailanddania.c@psu.ac.th (D.C.); 3 Biosignal Research Center for Health, Faculty of Science, Prince of Songkla University, Songkhla 90110, Thailand

**Keywords:** water avoidance stress, voiding spot analysis, urinary bladder dysfunction, bladder contraction, carbachol, ondansetron, anxiety-like behaviors, psychological stress

## Abstract

**Simple Summary:**

Psychological stress triggers an imbalance of neural and hormonal systems, leading to pathological conditions in many organ systems, especially the urinary bladder. Water avoidance stress (WAS) exposure for 10 days is a well-known rodent model that induces chronic psychological stress and impairs urinary bladder structure and function. However, the impact of WAS exposure in a different period apart from 10 days on anxiety-related behavior and urinary bladder structure and function remains unknown. Here, we investigated the effect of 1 day (acute), 10 days (chronic), and 28 consecutive days (prolonged) of WAS exposure and determined changes in anxiety-related behaviors, voiding patterns, mast cell numbers in bladder tissues, and bladder contractile properties that reflect pathological conditions from the stress exposure. Our study provides useful information on using WAS in an acute period (1 day) and prolonged period (28 days) on changes in anxiety-related behaviors and urinary bladder functions. The findings from this study are also essential for biomedical researchers to appropriately utilize the period of WAS exposure to investigate the effect of psychological stress-induced changes in the urinary bladders or other internal organs in a shorter or longer period than 10 days of WAS induction in a mouse model.

**Abstract:**

Repeated water avoidance stress (WAS) for 10 days is a common rodent model to mimic the effect of chronic psychological stress on urinary bladder dysfunction. However, it remains obscure whether changes in the stress exposure period impact urinary bladder impairment differently. Therefore, this study aimed to investigate the effect of 1 (acute), 10 (chronic), and 28 (prolonged) days of WAS on anxiety-related behavior, voiding pattern, urinary bladder mast cells, and bladder contractility in C57BL/6J male mice. Mice exposed to 1 and 10 days of WAS showed decreased unsupported rearing. A decreased total void area after 1 and 10 days of the WAS was observed, which was reversed in the 28-day-WAS group. There was an increased number of degranulated mast cells in the bladder of the 10-day-WAS group. The 1-day WAS exposure enhanced tonic contractile response to a muscarinic agonist, carbachol, which was reversed by 5-HT_3_ receptor antagonist pre-incubation. Interestingly, the 28-day WAS group showed a similar tonic contractile response to the control group. Our findings provide more insightful information about using 1-day WAS as an acute psychological stress model, and stress exposure longer than 10 days did not produce anxiety-like behavior and urinary bladder impairment.

## 1. Introduction

Psychological stress disrupts the balance of the body’s hormonal and neuronal systems, causing various chronic health issues [1]. Previous investigations have shown that psychological stress develops and worsens lower urinary tract symptoms, especially overactive bladder (OAB) syndrome and interstitial cystitis/painful bladder syndrome (IC/PBS) [2,3]. These pathological conditions of the urinary bladder significantly impact the quality of life, distressing various aspects of daily and social activities, including physical exercise, sleep, and sexual function [4]. Water avoidance stress (WAS) is a well-accepted animal model used to study the effects of psychological stress on urinary bladder and bowel functions in rodents [5]. Animals exposed to mild stressors repeatedly in the WAS model potentially resemble the daily stress pattern of humans [6]. Previous investigations utilized 10-day WAS exposure to induce urinary bladder dysfunction that is related to chronic psychological stress in rodents [7,8,9,10]. Female mice exposed to repeated WAS induced for 10 days showed increased plasma corticosterone, urinary frequency, and small urine spots but reduced total voided volume [10]. This is correlated with our previous investigation that reported a decrease in total voided area without changes in the total number of urine spots after WAS exposure for 10 days [8]. In addition, repeated WAS exposure showed visceral hypersensitivity during bladder filling and enhanced brain activity in areas associated with micturition, such as the pontine micturition center, periaqueductal grey, and prefrontal cortex [11].

Animals exposed to WAS for 10 days also developed bladder histopathological changes, i.e., urothelial damage and increased mast cell infiltration in the bladder tissues [8,12]. In addition to alterations in voiding behaviors and histological structure of the bladder, bladder tissues from 10-day WAS-induced mice showed increased spontaneous contractions and greater sensitivity to a muscarinic agonist, carbachol, suggesting changes in muscarinic response of the bladder in this model [8,10]. Our recent investigation also reported the contribution of 5-HT_3_ receptors using a 5-HT_3_ antagonist, ondansetron, in enhanced muscarinic response of the bladder in mice receiving WAS for 10 days [8]. Interestingly, a recent study noted partial recovery of bladder function in response to a muscarinic agonist after cessation of WAS exposure, but micturition frequency remained impaired [9]. Previous investigations utilized a 1-day WAS model as an acute stress of psychological stress [7,13]. Metabolic and transcriptome analyses revealed significant metabolic changes in the brains of mice exposed to acute WAS for 1 day [13]. Acute WAS induction for 1 day in rats has been reported to increase the volume of the first void and the number of voiding. Alterations in voiding in repeated WAS for 10 days could persist for approximately one month [7]. However, whether acute WAS for 1 day affects changes in anxiety-like behavior, affects urinary bladder contractile properties, or impacts bladder mast cell number remains elusive.

Even though previous investigations have reported the effect of WAS on urinary bladder dysfunctions, a limited number of studies have extended WAS beyond 10 days. McGonagle et al., 2012, reported that mice subjected to WAS 5 days a week for 4 weeks exhibited changes in voiding phenotypes, including increased voided volume and decreased urinary frequency [14]. However, this investigation did not report bladder contractile properties, mast cell number, and anxiety-related behaviors in this animal model. Given these limitations, further investigation into changes in urinary bladder functions and anxiety-related behaviors in mice exposed to WAS, both acute (1 day) and prolonged (28 days), is required. Therefore, our study aimed to investigate the effects of 1, 10, and 28 days of WAS exposure on anxiety-related behaviors, voiding patterns, mast cell counts in the bladder, and the role of 5-HT_3_ receptors in mediating the muscarinic response of bladder contractility. Findings from this investigation would increase the understanding of the effects of acute (1 day) and prolonged (28 days) WAS exposure and verify the experimental period of the utilization of WAS as a model to induce urinary bladder impairment related to psychological stress.

## 2. Materials and Methods

### 2.1. Animals

**Figure 1:**
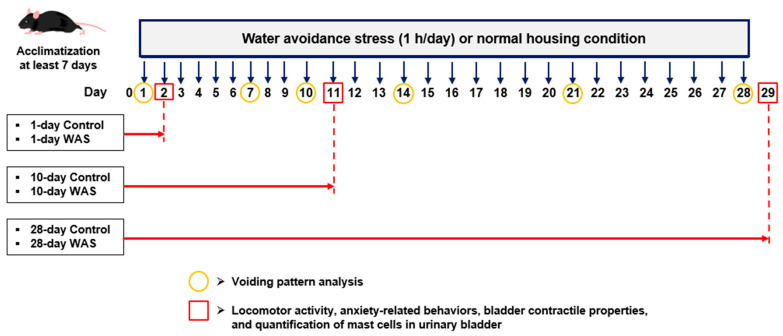
A scheme demonstrating the experimental timeline of this study.

All experimental protocols were approved by the Animals Ethical Committee, Prince of Songkla University, Thailand (protocol code 2023-SCI05-010). Adult male C57Bl/6NJcl mice (8–10 weeks old) were purchased from Nomura Siam International Co., Ltd., Bangkok, Thailand. Mice were housed in a 12:12 h light-dark (temperature at 25 ± 2 °C) controlled room. All mice were acclimatized for at least one week and maintained individually in the cage with freely available water and a standard diet (Perfect Companion Group Co., Ltd., Samut Prakan, Thailand). Mice were randomly allocated into six groups: 1-day Control, 10-day Control, 28-day Control, 1-day WAS, 10-day WAS, and 28-day WAS. The body weights of the animals were measured daily throughout the water avoidance stress timeline. The experimental timeline for this study is depicted in Figure 1.

### 2.2. Water Avoidance Stress (WAS) Protocol

Exposure to WAS protocol was performed in this study as described previously [8]. A standard polypropylene box with dimensions of 43 cm in length, 29 cm in width, and 20 cm in height was filled with room-temperature water up to 2 cm below the top of a cylindrical platform. The platform (6 cm length, 6 cm width, and 11 cm height) was placed at the center of the box. Mice in the WAS groups (1-day WAS, 10-day WAS, and 28-day WAS) were positioned on the platform for one hour daily for 1 day, 10 days, and 28 consecutive days from 10 a.m. to 11 a.m. Meanwhile, the control groups (1-day control, 10-day control, and 28-day control) were maintained in their cages individually.

### 2.3. Voiding Spot Analysis

Voiding spot analysis was conducted after the WAS procedure on days 1, 7, 10, 14, 21, and 28. Mice were placed on a wire mesh in a standard cage with the floor lined with filter paper (Whatman™ Grade 1, Cat No. 17023133) for four hours. To prevent urine spots from being diluted and spread by dripping water, mice were subjected to water deprivation but still had free access to food during the experimental period. The filter paper was dried for 24 h, and the urine spots were photographed under UV light. The number of urine spots, total voided area, and proportion of small (size 0.4–5.99 cm^2^) and large (size ≥ 6.00 cm^2^) urine spots were quantified using ImageJ Software version 1.54d.

### 2.4. Open Field Test (OFT)

One day after the end of the WAS period, mice from all groups underwent the open field test (OFT) to assess their locomotor activity and anxiety-like behavior as described previously [15]. Briefly, mice were placed in a white plastic box (15-inch length, 12-inch width, and 12-inch height) and were allowed to freely explore the open field box for 15 min. The behaviors and movements of mice were recorded by using a video camera positioned at the top of the box. The OptiMouse MATLAB software version R2023a was used to detect the locomotor activity of mice by contrasting the black color of the animals’ bodies with the white background of the box. Additionally, anxiety-like behaviors were observed on the following criteria: (1) grooming was identified as mice rubbing or licking their paws, head, or body for more than 2 s; (2) rearing was defined as being of 2 types, namely, (2.1) supported rearing, which was scored when the mice stood on their hind legs and leaned against the walls of the box, and (2.2) unsupported rearing, which was noted when the mice stood on their hind legs without support from the wall.

### 2.5. Organ Bath Studies

One day after finishing the WAS or control experimental protocols, the mice were anesthetized with sodium pentobarbital (70 mg/kg) and underwent laparotomy. The urinary bladder was collected and weighed. Thereafter, the urinary bladder was cut and trimmed in a rectangular shape (~0.5 × 1 cm). The tissues were placed in a tissue chamber containing 20 mL of oxygenated Krebs solution (composition in mM: NaCl 119, glucose 11.1, KCl 4.5, MgSO_4_ 2.5, KH_2_PO_4_ 1.2, NaHCO_3_ 25, CaCl_2_ 2.5) maintained at 37 °C. The tissues were pre-tensioned to approximately 2 g and allowed to equilibrate for 20 min before starting the experiment.

Before assessing bladder contractile properties of the muscarinic agonist, a high concentration of KCl (80 mM) was used to evaluate the tissue viability. Subsequently, the tissues were tested for their contractile response to cumulative concentrations of the muscarinic agonist carbachol (CCh) (Sigma-Aldrich, Darmstadt, Germany) at 0.1, 0.3, 1, 3, 10, and 30 μM, with 5 min intervals between each concentration. Furthermore, the contribution of 5-HT_3_ receptors in mediating changes in bladder contraction in the stress groups (1-day WAS and 28-day WAS) was investigated by pre-incubating the tissues with a 5-HT_3_ receptor antagonist, ondansetron (30 nM), for 20 min before conducting dose–response studies with CCh. Tonic contraction of the tissues was recorded using the PowerLab^®^ System (AD Instruments, Bella Vista, NSW, Australia) and analyzed with LabChart version 7.0 (AD Instruments, Chalgrove, UK).

### 2.6. Toluidine Blue Staining for Mast Cell Quantification

Bladder tissues from all groups were fixed in 10% formalin and dehydrated in a series of ethanol solutions (70%, 95%, and 100% ethanol). After dehydration, the tissues were embedded in paraffin and sectioned at a thickness of 5 µm. The tissues were stained with 1% toluidine blue (Merck, Darmstadt, Germany) to determine non-degranulated (no granular extrusion) and degranulated mast cells (extruded granules). Three tissue sections from each mouse were randomly determined. The total number of mast cells for each mouse was counted and averaged from these three distinct sections. Additionally, the percentage of non-degranulated and degranulated mast cells in the urinary bladder was calculated. The identification and counting of mast cells were examined using a blinded approach with microscopic visual fields (Optika, B-383PLi, Ponteranica BG, Italy) at 10× and 40× magnifications.

### 2.7. Statistical Analysis

Data were expressed as mean ± standard deviation (SD). The statistical significance between groups was determined using an Unpaired *t*-test. Tonic contractions in response to carbachol were analyzed using one-way ANOVA with Dunnett’s multiple comparisons. Differences were considered statistically significant at *p* < 0.05 using GraphPad Prism 9.0 (GraphPad Software, San Diego, CA, USA).

## 3. Results

### 3.1. Changes in Locomotor Activity and Anxiety-Related Behavior

We observed no significant differences in locomotor activities, including average speed, total distance traveled, and total time spent in the center zone, between the control and WAS groups in mice exposed to the WAS protocol for 1, 10, and 28 days (Unpaired *t*-test, *p* > 0.05) (Table 1).

**Figure 2:**
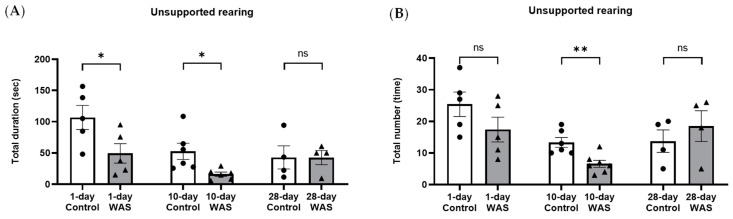

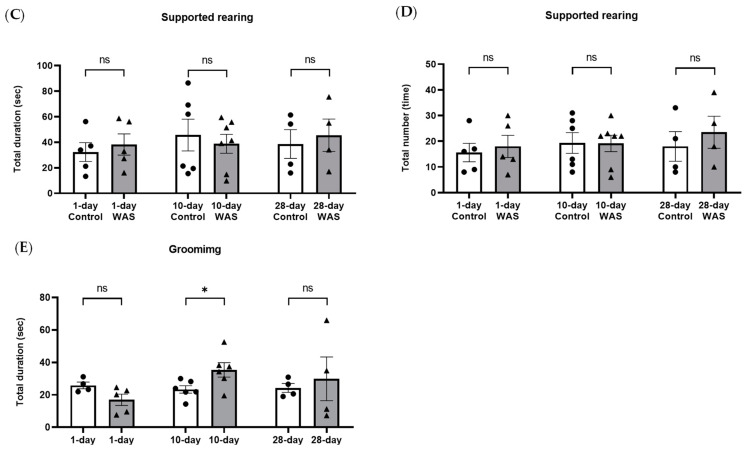
Changes in anxiety-related behavior of control and water avoidance stress (WAS) on days 1, 10, and 28. The total duration (**A**) and the total number (**B**) of unsupported rearing. The total duration (**C**) and the total number (**D**) of supported rearing. The total duration of grooming behavior (**E**). (* *p* < 0.05, ** *p* < 0.01, Unpaired *t*-test, ns = not significant, N = 4–7 in each group.)

In addition, anxiety-related behavior was observed in mice during the open field test. Mice exposed to WAS for 1 day showed a significant reduction in the total duration of unsupported rearing (WAS: 49.41 ± 34.70 s vs. Control: 106.80 ± 43.00 sec, Unpaired *t*-test, * *p* < 0.05) (Figure 2A) but no significant difference in the total number of unsupported rearing (Figure 2B) and the total duration of grooming behaviors compared with the control group (Unpaired *t*-test, *p* > 0.05) (Figure 2E). Mice exposed to WAS for 10 days also showed a significant decrease in the total duration of unsupported rearing (WAS; 16.17 ± 8.48 s vs. Control; 52.74 ± 31.61 s, Unpaired *t*-test, * *p* < 0.05) (Figure 2A) and the total number of unsupported rearing compared with the control group (WAS; 6.57 ± 2.94 times vs. Control; 13.33 ± 3.83 times, Unpaired *t*-test, ** *p* < 0.01) (Figure 2B). Meanwhile, the total duration of grooming behaviors showed a significant increase in the 10-day WAS group compared to 10-day control group (WAS; 35.40 ± 10.82 times vs. Control; 23.38 ± 5.55 times, Unpaired *t*-test, * *p* < 0.05) (Figure 2E).

Interestingly, mice exposed to WAS for 28 days did not show a significant difference in the total duration (Figure 2A) and the total number (Figure 2B) of unsupported rearing behaviors, as well as the total duration of grooming behavior (Figure 2E), compared with the control group (Unpaired *t*-test, *p* > 0.05). There were no significant differences in the total duration (Figure 2C) and the total number (Figure 2D) of supported rearing behavior between the control and WAS groups on days 1, 10, and 28 (Unpaired *t*-test, *p* > 0.05).

### 3.2. Body Weight and Bladder Weight

There was no significant difference in the body weight of mice between the control and WAS groups of 1, 10, and 28 days after WAS exposure (Unpaired *t*-test, *p* > 0.05) (Table 2). In addition, bladder weights and normalized bladder weight with body weight showed no significant changes between the control and the WAS groups at the same exposed time (Unpaired *t*-test, *p* > 0.05) (Table 2).

### 3.3. Urine Pattern Analysis

**Figure 3:**
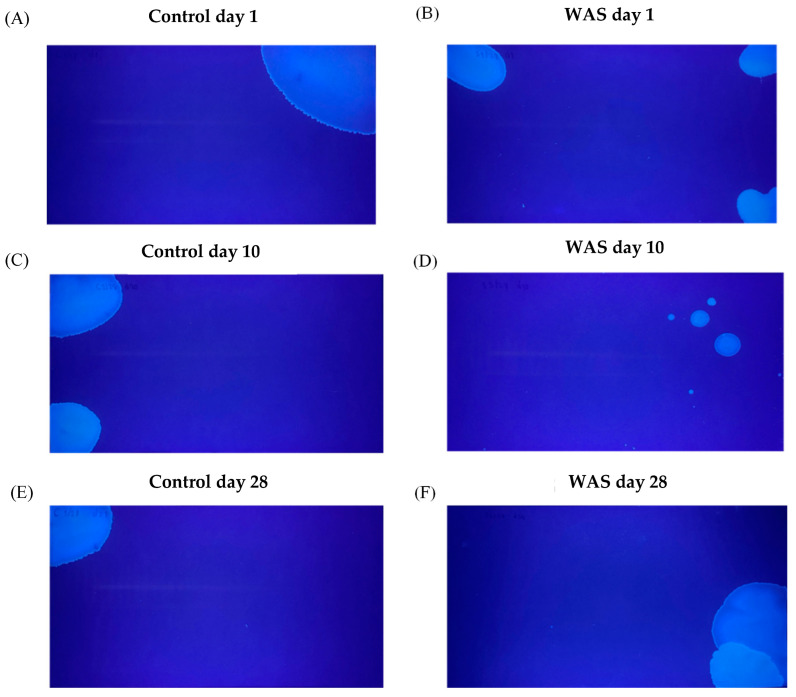
Representative images of urine patterns on filter papers on days 1, 10, and 28 of control (**A**,**C**,**E**) and water avoidance stress (WAS) (**B**,**D**,**F**) groups.

**Figure 4:**
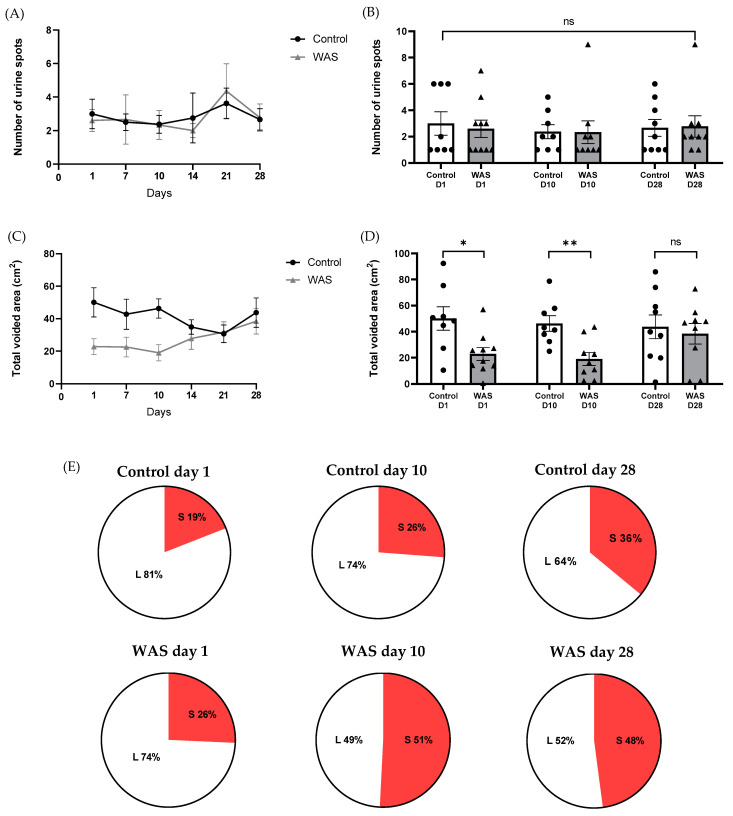
Changes in the number of urine spots (**A**,**B**) and total voided area (**C**,**D**) from voiding spot analysis of the control and water avoidance stress (WAS) groups on days 1, 10, and 28. (* *p* < 0.05, ** *p* < 0.01, Unpaired *t*-test, ns = not significant, N = 8–10 in each group). Pie graphs representing the proportion of large urine spot (L) (size ≥ 6.00 cm^2^) and small spot size (S) (size 0.4–5.99 cm^2^) from the voiding spot analysis of the control and water avoidance stress (WAS) groups on days 1, 10, and 28 (**E**) (N = 8–10 in each group).

Representative images of urine voiding patterns on filter paper on days 1, 10, and 28 from the 28-day control and 28-day WAS groups are shown in Figure 3. The number of urine spots and the total voided area were determined on days 1, 7, 10, 14, 21, and 28 of WAS induction (Figure 4A,C). The trends of voided area were reversed and not different from the control group on days 14, 21, and 28 of the stress induction (Figure 4C). On day 1, the total voided area, indicating urine volume, was significantly reduced in the WAS group compared to the control group (WAS; 22.85 ± 15.50 cm^2^ vs. Control; 50.12 ± 25.50 cm^2^, Unpaired *t*-test, * *p* < 0.05) (Figure 4D). On day 10, the total voided area of the WAS group was significantly decreased compared to the control group (WAS; 19.03 ± 14.99 cm^2^ vs. Control; 46.3 ± 16.87 cm^2^, Unpaired *t*-test, ** *p* < 0.01) (Figure 4D). Interestingly, the WAS group showed no significant changes in the total voided area on day 28 compared to the control group (WAS; 38.42 ± 23.67 cm^2^ vs. Control; 43.74 ± 27.34 cm^2^, Unpaired *t*-test, *p* > 0.05) (Figure 4D). There was no significant difference in the number of urine spots between the control and WAS groups on days 1, 10, and 28 (Unpaired *t*-test, *p* > 0.05) (Figure 4A,B). Moreover, proportions of large urine spots (L) and small urine spots (S) were analyzed (Figure 4E). There was a trend of increasing proportion of the small urine spots in the WAS compared to the control on day 1 after the WAS protocol (Control 19% vs. WAS 26%), day 10 (Control 26% vs. WAS 51%), day 28 (Control 36% vs. WAS 48%).

### 3.4. Mast Cell Number in Bladder Tissues

**Figure 5:**
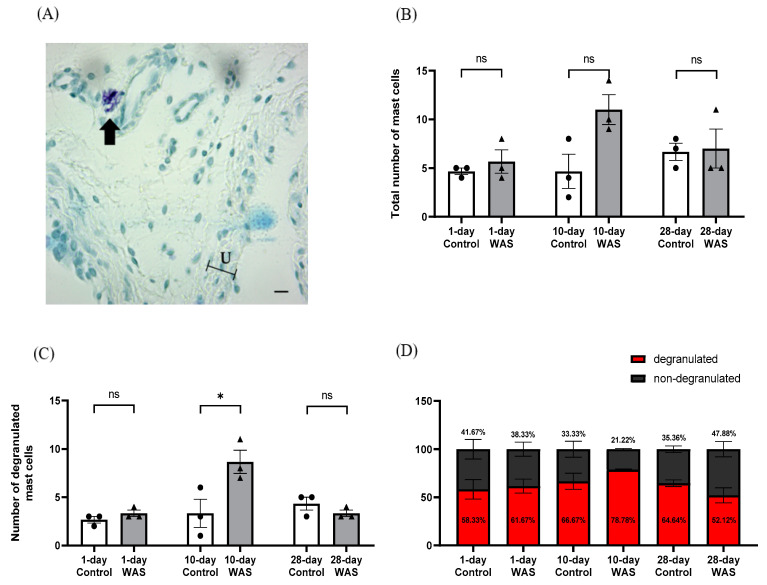
Representative images of degranulated mast cells (**A**) in the urinary bladder as indicated with the arrows. Bar graphs representing the total number of mast cells (**B**) and the number of degranulated mast cells (**C**). The stacked bar graph represents percentages of degranulated and non- degranulated mast cells in the bladder tissues of the control and water avoidance stress (WAS) groups on days 1, 10, and 28 (**D**). Scale bar = 10 µm, 40× magnification, U = Urothelium (* *p* < 0.05, Unpaired *t*-test, ns = not significant, N = 3 in each group).

The number of mast cells in bladder tissues was determined in all groups. Representative images of degranulated mast cells are shown in Figure 5A. The total number of degranulated mast cells of the 10-day WAS group was significantly increased compared to the control group (WAS; 8.67 ± 2.08 vs. Control; 3.33 ± 2.52, Unpaired *t*-test, * *p* < 0.05) (Figure 5C). The WAS group showed no significant changes in the total number of degranulated mast cells of the 1-day and 28-day WAS groups compared to the control groups (Unpaired *t*-test) (Figure 5C). There were no differences in the total number of mast cells (Figure 5B) and the percentage of degranulated and non-degranulated mast cells (Figure 5D) in the bladder tissues between the WAS and control groups on days 1, 10, and 28 (Unpaired *t*-test).

### 3.5. Tonic Contractile Properties of Bladder Tissues in Response to Carbachol (CCh) and Ondansetron Pre-Incubation

**Figure 6:**
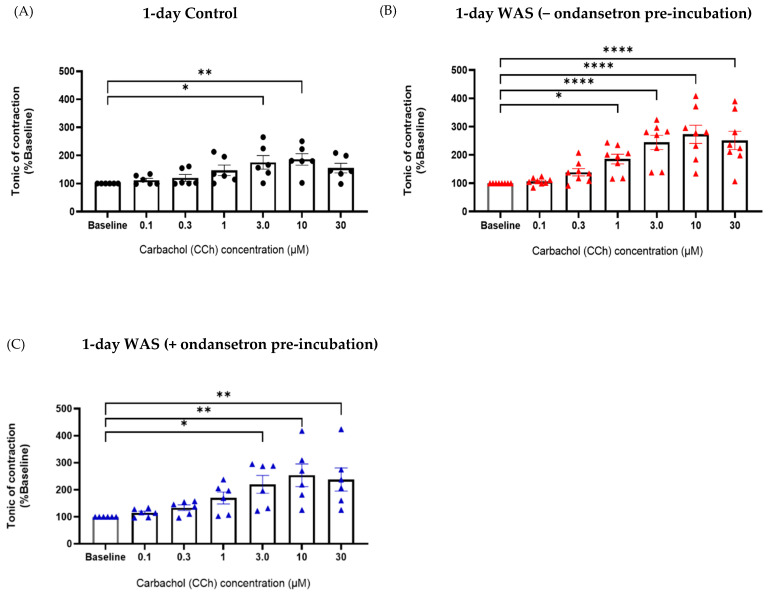
Tonic contraction of bladder strips from the control (**A**) and 1-day WAS groups in response to 0.1, 0.3, 1.0, 3.0, 10, and 30 µM carbachol with (**C**) or without ondansetron pre-incubation (**B**) (* *p* < 0.05, ** *p* < 0.01, **** *p* < 0.0001, One-way ANOVA with Dunnett’s multiple comparison vs. baseline, N = 6–8).

The viability of the bladder tissues was determined using an application of a high concentration of KCl (80 mM). We observed no significant difference in tonic contraction in response to KCl between the control and WAS groups in both 1-day and 28-day WAS groups. The 1-day control group exhibited a significant increase tonic contraction in response to CCh at 3.0 µM (175.3 ± 59.80% baseline, One-way ANOVA with Dunnett’s multiple comparisons, * *p* < 0.05) (Figure 6A), while the 1-day WAS group showed a tonic contractile response to CCh at 1.0 and 3.0 µM (185.7 ± 48.18% baseline and 244.5 ± 71.08% baseline, respectively, One-way ANOVA with Dunnett’s multiple comparisons, * *p* < 0.05) (Figure 6B).

**Figure 7:**
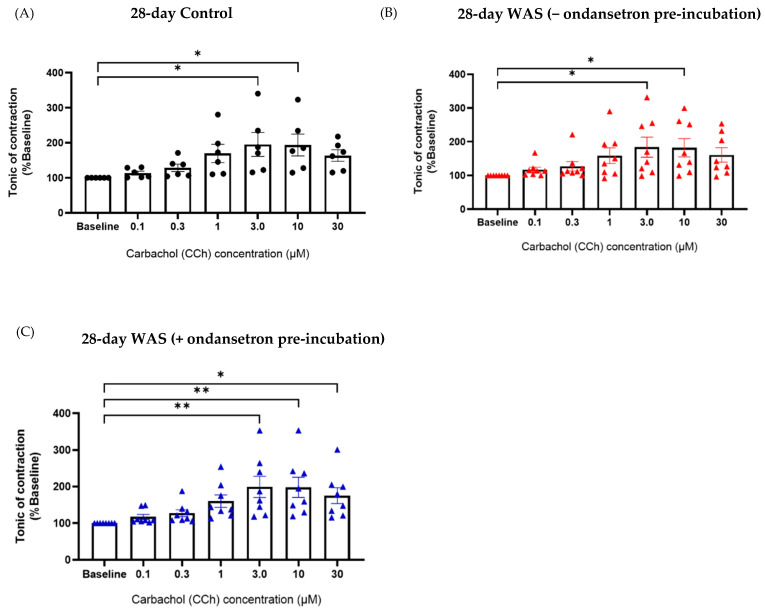
Tonic contraction of bladder strips from the control (**A**) and 28-day WAS groups in response to 0.1, 0.3, 1.0, 3.0, 10, and 30 µM carbachol with (**C**) or without ondansetron pre-incubation (**B**) (* *p* < 0.05, ** *p* < 0.01, One-way ANOVA with Dunnett’s multiple comparisons vs. baseline, N = 6–8).

Interestingly, pre-incubation with ondansetron (30 nM) before CCh stimulation reversed the response of CCh at 3.0 µM for the WAS group (WAS with ondansetron pre-incubation; 220 ± 81.17% baseline, One-way ANOVA with Dunnett’s multiple comparisons, * *p* < 0.05) (Figure 6C). In contrast, the bladder tissues of the 28-day WAS and 28-day control groups showed a similar tonic contractile response to CCh as the control group at 3.0 µM (184.1 ± 84.34% baseline and 195.3 ± 83.67% baseline, respectively) (Figure 7A,B). Ondansetron pre-incubation before CCh stimulation did not reverse tonic contractile response in the 28-day WAS group (198.9 ± 81.59% baseline, One-way ANOVA with Dunnett’s multiple comparisons, *p* > 0.05) (Figure 7C).

## 4. Discussion

The present study investigated changes in anxiety-related behavior, voiding pattern, mast cells in the bladder tissues, and contractile response to muscarinic stimulation via 5-HT_3_ modulation in mice exposed to acute WAS exposure (1 day), chronic WAS (10 days), and prolonged WAS exposure (28 days). The mice that received acute 1-day and 10-day WAS exposure showed a decrease in the duration of unsupported rearing behavior, indicating the signs of stress in rodents. The 10-day WAS group also showed a decrease in the total number of unsupported rearing with increased total duration of grooming. Our results suggest that mice exposed to WAS for 1 day and 10 days showed anxiety-like behavior. Our finding is consistent with a recent investigation that reported higher levels of plasma corticosterone in mice exposed to WAS for 1 day and 10 days [10,13]. We observed no significant difference in locomotor activity including averaged speed, total distance traveled, and time spent in the center zone of 1,10, and 28 days of WAS exposure compared to the control groups. This is concurrent with our previous investigation that observed no significant changes in locomotor activity in 10-day WAS mice, while the WAS-induced mice showed a significantly decreased number of unsupported rearing behaviors [15]. A previous study reported that unsupported rearing behavior in rodents could reflect emotional stress [16]. Interestingly, 28 consecutive days of WAS exposure did not exhibit significant changes in either locomotor activity or anxiety-like behaviors, i.e., rearing and grooming when compared to the control group. This might be explained by the stress adaptive mechanism to homotypic stressors. It is plausible that the sympathoadrenomedullary (SAS) system becomes resistant to the stressor in the condition of chronic/prolonged stress exposure. Our finding is related to previous investigations that reported that several weeks of immobilization leads to lesser response to homotypic stimulus and increased baseline levels of catecholamines [17,18]. However, stress hormonal markers, i.e., corticosterone, should be determined to confirm the stress adaptation in the 28-day WAS group.

We consecutively performed voiding pattern analysis of mice that received WAS on days 1, 7, 10, 14, 21, and 28 of the protocol. We observed a significant decrease in total voided area (urine volume) on day 1 and day 10 in the WAS groups. The possible mechanism of reduced urine volume after 1 day and 10 days of WAS protocol might derive from stress-increased sympathetic activity and catecholamine release. Previous evidence in patients with painful bladder syndrome (PBS) showed high levels of norepinephrine levels in the urine [19]. However, our finding is in contrast with the previous investigation showing an increase in urine volume after 1 day of WAS in female mice [10]. The different urine areas after 1 day WAS may derive from sex differences, which might be influenced by the alternation of sex hormones. Previous studies have also reported the influence of sex differences in water avoidance stress-induced urinary bladder dysfunction [10,14,20]. The reversed changes in voiding pattern after 28 days of WAS might derive from a stress adaptative mechanism, which might derive from SAS and/or HPA axis mechanisms [21]. We did not detect changes in the total number of urine spots in mice that received prolonged WAS on days 1, 10, and 28 after the WAS protocol. However, the proportion of small urine spots tends to increase from 1-day WAS (26%) to 10-day WAS (51%). The 28-day WAS showed percentages of small urine spots similar to the 10-day WAS exposure (48%). Changes in a small proportion of small urine spots suggest there might be dysregulation of detrusor muscle and/or neural control of the urinary bladder, leading to uncontrolled voiding as a sign of urinary frequency. The voiding patterns of day 1 and day 10 of WAS protocol in this study were related to a previous clinical report on the relationship between psychological stress and voiding dysfunctions, e.g., IC/PBS, overactive bladder, and urinary retention [22,23,24].

Mast cells are involved in the storing and release of various inflammatory mediators and neuropeptides, such as histamine, prostaglandins, leukotrienes, cytokines, and serotonin (5-HT) [12,25]. An increasing amount of evidence indicates that stress exacerbates inflammatory disease and increases mast cell activation syndrome [26,27]. Previous studies reported that stress-induced activation of bladder mast cells can exacerbate bladder hypersensitivity and inflammation, contributing to the symptoms in the conditions of interstitial cystitis/bladder pain syndrome (IC/BPS) [7,28]. In this study, we investigated the number of degranulated mast cells in the bladder tissues of mice exposed to acute (1 day), chronic (10 days), and prolonged (28 days) WAS. The bladder tissues of the 10-day WAS group had a significant increase in the number of degranulated mast cells compared to the control group. This is consistent with a previous investigation that demonstrated an elevated proportion of degranulated mast cells in the bladder tissues of male mice exposed to 10 days of WAS [8]. Additionally, rats subjected to 10 days of WAS exhibited mast cell infiltration in the bladder tissues, which is partially regulated by alpha-1 adrenergic receptors [12]. The related mechanism of psychological stress-induced mast cell infiltration in the bladder wall might be due to activating proinflammatory effects via stress-induced corticotropin-releasing hormone (CRH) from the hypothalamus. A clinical report showed that patients with psychological stress have high levels of CRH in the serum and mastocytosis conditions [29,30]. However, we observed no significant difference in the numbers of total mast cells (degranulated and non-degranulated mast cells) in the bladder wall of 1 day and 28 days of WAS-exposed groups when compared to the controls, which is also related to changes in the voiding profile from day 1 to day 28 of the WAS protocol. These results indicate that exposure to WAS for 10 consecutive days might be the most effective stress exposure time to induce both histological and functional changes in the urinary bladder.

Previous studies have primarily focused on the effects of 10-day repeated water avoidance stress [7,8,9,10,11,12]. However, the impact of different stress periods, including acute stress (1 day) and prolonged stress (28 days), on bladder contractile properties remains to be characterized. Our study shows that mice exposed to 1 day of WAS displayed a significant increase in tonic contraction in response to carbachol at 1.0, 3.0, 10, and 30 μM, whereas the control group exhibited a biphasic concentration response, with a significant increase at 3.0 and 10 μM of carbachol. These results suggest that acute WAS-induced mice exhibited heightened responsiveness to muscarinic stimulation in bladder tissue compared to the control group. This observation aligns with previous studies conducted under 10-day WAS conditions. Our recent study on 10-day WAS in male mice demonstrated enhanced tonic contractions in response to carbachol stimulation in isolated bladder tissues compared to unstressed mice [8]. Moreover, female mice subjected to a 10-day WAS protocol exhibited bladder overactivity and enhanced phasic activity following exposure to carbachol [10]. Another study found that cold stress-induced detrusor hyperactivity in the urinary bladder was mediated by elevated expression of muscarinic M_3_ receptors in type 2 diabetes mellitus-induced rats [31]. Furthermore, mice exposed to WAS for 10 days showed a significant increase in corticosterone levels and bladder overactivity phenotypes. Treatment with the muscarinic receptor antagonist solifenacin reduced WAS-induced bladder overactivity and restored corticosterone levels [32]. A previous study demonstrated that treatment with a glucocorticoid receptor-specific antagonist (CORT108297) prevented the increase in colonic tissue acetylcholine levels and inhibited in vivo colonic motility in 4-day WAS mice [28]. This information might suggest that acute WAS (1 day) may be regulated via the SAS and HPA axes, leading to bladder hypercontractility, which involves alteration in muscarinic signaling in the bladder tissues.

The interaction between muscarinic and serotonergic signaling via 5-HT_3_ receptors in modulating the increased bladder contractile properties was then further examined. Increased tonic response to CCh in the 1-day WAS group was mitigated by pre-incubation with the 5-HT_3_ receptor antagonist, ondansetron. This finding corresponds to our earlier study that ondansetron pre-incubation (30 nM) attenuated carbachol-induced tonic contraction in mice exposed to a 10-day WAS protocol [8]. There is evidence reported that 5-HT_3_ receptors might regulate acetylcholine release during 5-HT-potentiated detrusor contraction induced by electrical field stimulation [33]. This information suggests that acute WAS exposure may promote bladder hypercontraction, which derives from the modulation of muscarinic signaling via 5-HT_3_ receptors in the urinary bladder.

Interestingly, the prolonged 28-day WAS group showed increased tonic contractile response to carbachol at the concentration of 3.0 μM similar to the control group. Additionally, bladder tissues of the 28-day WAS group with ondansetron pre-incubation before CCh stimulation showed the same tonic contractile response as the control and the 28-day WAS without ondansetron incubation. Therefore, this result indicates that mice exposed to 28-day WAS exhibited reversed high sensitivity of bladder contractility to muscarinic stimulation compared to 1-day and 10-day WAS groups. The reversed changes in unsupported rearing behavior, reduced urine volume, degranulated mast cells, and contractile response to a muscarinic stimulation might be explained by a stress adaption to homotypic stressor via increased basal levels of hormones in the stress response, i.e., catecholamines [17,18]. One of the limitations of this study is that hormonal markers, i.e., corticosterone, should be further monitored to confirm stress adaptation in this model. Future investigation is essential to verify the direct contribution and underlying mechanism of the HPA axis or sympathoadrenomedullary (SAS) system in prolonged WAS (longer than 10-day period) to confirm the stress adaptation mechanisms in the WAS model.

## 5. Conclusions

Acute exposure to WAS for 1 day decreased the total duration of unsupported rearing behavior similar to repeated WAS for 10 days. Mice that received 1-day WAS had reduced voided area and increased bladder contractile response to muscarinic stimulation, which was reversed by a 5-HT_3_ receptor antagonist, ondansetron. Prolonged WAS exposure for 28 days did not show anxiety-like behaviors and reversed impaired voiding pattern and bladder hypercontractility. This study provides extensive information on changes in anxiety-like behaviors and urinary bladder impairment and related mechanisms in mediating bladder hypercontractility in the acute (1 day) WAS model. WAS exposure longer than 10 days did not worsen or impact anxiety-related behavior and urinary bladder dysfunction.

## Figures and Tables

**Table 1:** Locomotor activity of mice exposed to water avoidance stress for 1, 10, and 28 days.

Parameters	Acute Period (1 Day)		Chronic Period (10 Days)		Prolonged Period (28 Days)	
	1-Day Control (N = 5)	1-Day WAS (N = 5)	10-Day Control (N = 6)	10-Day WAS (N = 7)	28-Day Control (N = 4)	28-Day WAS (N = 4)
Averaged speed (cm/s)	4.112 ± 0.93	3.931 ± 0.64	3.858 ± 1.11	4.373 ± 1.01	3.490 ± 0.84	4.661 ± 1.02
Total distance traveled (cm)	2465 ± 554.70	2335 ± 349.50	2313 ± 661.90	2623 ± 632.10	2092 ± 504.30	2795 ± 609.20
Time spent in the center zone (sec)	142.3 ± 62.89	166.7 ± 111.60	186.4 ± 138.60	108.7 ± 31.27	182.0 ± 140.60	164.8 ± 121.10

**Table 2:** Body weight and bladder weight of the animals in all groups.

Parameters	Acute Period (1 Day)		Chronic Period (10 Days)		Prolonged Period (28 Days)	
	1-Day Control (N = 9)	1-Day WAS (N = 11)	10-Day Control (N = 10)	10-Day WAS (N = 11)	28-Day Control (N = 11)	28-Day WAS (N = 10)
Body weight (g)	27.30 ± 2.854	25.90 ± 3.335	26.73 ± 1.890	26.89 ± 3.165	26.49 ± 1.342	26.23 ± 2.031
Bladder weight (g)	0.034 ± 0.004	0.036 ± 0.010	0.034 ± 0.005	0.036 ± 0.008	0.034 ± 0.009	0.037 ± 0.006
% Bladder weight/body weight	0.127 ± 0.018	0.140 ± 0.028	0.128 ± 0.020	0.133 ± 0.028	0.130 ± 0.034	0.139 ± 0.020

Body weight and bladder weight were determined on the same day as the organ bath studies.

## Data Availability

Data are contained within the article.
